# The complete chloroplast genome of *Dipterocarpus turbinatus* Gaertn. F

**DOI:** 10.1080/23802359.2019.1677192

**Published:** 2019-10-18

**Authors:** Xiaotong Ci, Jinyu Peng, Chen Shi, Zeli Zhu, Nianhui Cai, Anan Duan, Dawei Wang

**Affiliations:** a Key Laboratory for Forest Resources Conservation and Utilization in the Southwest Mountains of China Ministry of Education, Southwest Forestry University, Kunming, China;; b Key Laboratory for Forest Genetic and Tree Improvement and Propagation in Universities of Yunnan Province, Southwest Forestry University, Kunming, China

**Keywords:** *Dipterocarpus turbinatus* Gaertn. F., chloroplast genome, phylogenetic analysis

## Abstract

*Dipterocarpus turbinatus* Gaertn. F., naturally distributes in Southern China, which is an elite natural tree with high economic and medicinal value. In study, all chloroplast (cp) genome of *Dipterocarpus turbinatus* Gaertn. F. was assembled and characterized based on Illumina pair-end sequencing data. The complete chloroplast genome length was 152,279 bp. It contained a large (LSC, 83,862 bp) and a small (SSC, 20,215 bp) single copy region, separated by a pair of inverted repeats of 24,101 bp (IRs). The overall GC content of genome was 37.3%, the corresponding values of LSC, SSC, and IR regions were 35.3, 31.6, and 43.2%, respectively. There were 128 genes in the genome including 84 protein-coding genes, 36 tRNA genes, and 8 rRNA genes. Among all genes, 14 genes contain a single intron and 1 gene has two introns. The result showed that *Dipterocarpus turbinatus* Gaertn. F. was closely related to *Vatica mangachapoi.*

*Dipterocarpus turbinatus* Gaertn. F. is a Dipterocarpaceae family evergreen macrophanerophytes which is widely distributed in Southeast Asia (Wang et al. 2010). It is a precious timber species for cultivating large diameter timber in Yunnan China (Wang et al. 1991). The resin secreted by the trunk is a raw material for the preparation of borneol and aromatic oil (Ren and Zhou 2007). It can be used as a medicinal herb with heat-clearing, anti-inflammatory (Huda et al. 2006). Mining its genetic information will help to study the species diversity and evolutionary relationships of this species.

Chloroplast genomes are widely used in the biology and function of organelles (Dong et al. 2013). So far, complete chloroplast genomes of several species from the Dipterocarpaceae family have been studied and deposited at the GenBank database, such as *Vatica mangachapoi* (Wang et al. 2018) and *Neobalanocarpus heimii* (Lee et al. 2019). However, the plastome of *Dipterocarpus turbinatus* Gaertn. F. has not been reported. In this paper, we assembled and characterized the complete chloroplast genome sequence of *Dipterocarpus turbinatus* Gaertn. F. employing the high-throughput sequencing approaches. It will provide benefits for further molecular studies on taxonomic and phylogenomic resolution in Theoideae order

The fresh leaves of *Dipterocarpus turbinatus* Gaertn. F. were collected from XiShuangBanNa Tropical Botanical Garden, Chinese Academy of Sciences (Yunnan, China; geospatial coordinates: 101°25′73″E, 21°41′96″N; Altitude: 570 m). The total genomic DNA was extracted by using the Magnetic beads plant genomic DNA preps Kit (TSINGKE Biological Technology, Beijing, China). The DNA samples (DTGF. 1) were stored at the Key Laboratory for Forest Resources Conservation and Utilisation in the Southwest Mountains of China Ministry of Education, Southwest Forestry University, Kunming, China.

The genome skimming sequencing was performed with 150 bp paired-end (PE) reads on the Illumina HiSeq 2000 platform. In total, approximately, 5.2 G high-quality clean reads were obtained. Then, using Geneious R8 (Biomatters Ltd, Auckland, New Zealand), assembled and performed the annotation of the complete cp genome. Finally, the chloroplast DNA sequence with complete annotation information was deposited at GenBank database under the accession number MN 444783.

The complete length of *Dipterocarpus turbinatus* Gaertn. F. cp genome was 152,279 bp, comprising of a large single copy region (LSC with 83,862 bp) and a small single copy region (SSC with 20,215 bp), which were separated by a pair of inverted repeats (IRs with 24,101 bp). The overall GC content of genome was 37.3%, the GC content of the LSC (35.3%), and SSC (31.6%) regions was relatively low than that of the IR regions (43.2%). A total of 128 functional genes were contained in the cp genome, including 84 protein-coding genes (PCG), 36 tRNA genes, and 8 rRNA genes. There were 17 genes that had two copies (genes in IR region), which included 6 PCG genes (ndhB, rpl2, rpl23, rps7, ycf2, and ycf15), 7 tRNA genes (trnA-UGC, trnI-CAU, trnI-GAU, trnLCAA, trnN-GUU, trnR-ACG, and trnV-GAC), and all 4 rRNA species (rrn4.5, rrn5, rrn16 and rrn23). Additionally, among all genes, 12 genes contain a single intron, of which 9 (clpP, ndhB, ndhB, petB, petD, rpl2, rpoC1, rps12, rps16) belong to PCG genes, 5 (rnA-UGC, trnI-GAU, trnK-UUU, trnL-UAA; trnV-UAC) belong to tRNA genes and 1 gene (ycf3) have two introns.

To uncover the phylogenetic relationship of *Dipterocarpus turbinatus* Gaertn. F. in Theoideae family, the other 19 related complete chloroplast genomes were collected for further analysis. The alignment of all cp genomes was done by the MAFFT version 7 software (Katoh and Standley 2013), which was analyzed by IQ-TREE 1.5.5 (Nguyen et al. 2015) under the GTR + F+R3 nucleotide substitution model (Kalyaanamoorthy et al. 2017). Finally, the phylogenetic analysis based on maximum likelihood (ML) tree was inferred with 1000 bootstrap replicates. The result showed that *Dipterocarpus turbinatus* Gaertn. F. appeared to be closely related to *Vatica mangachapoi* (Figure 1). In conclusion, complete cp genome of *Dipterocarpus turbinatus* Gaertn. F. is decoded for the first time, which will also provide essential DNA molecular data for further biological analysis.

**Figure 1.**
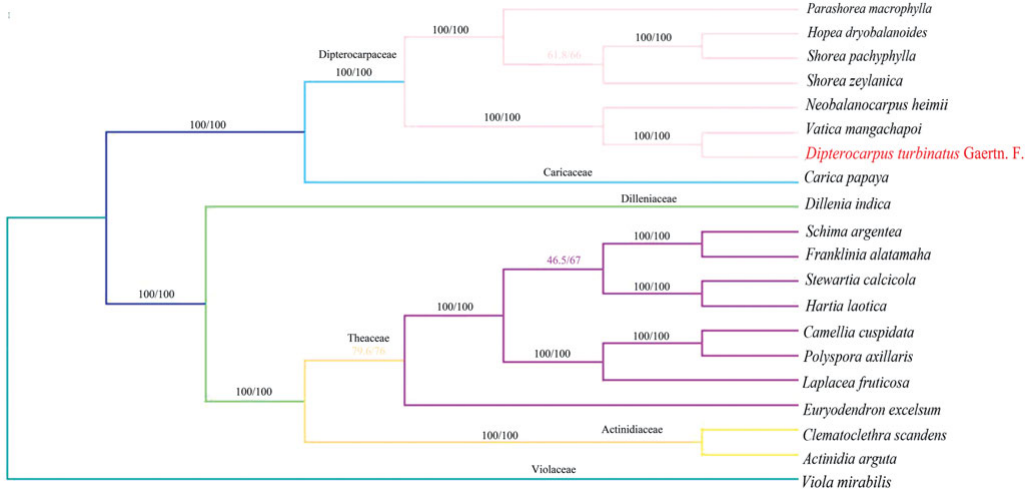
Phylogenetic relationship among 19 complete chloroplast genomes of Theoideae family. Bootstrap support values are given at the nodes. Chloroplast genome accession number used in this phylogeny analysis: *Parashorea macrophylla: MH791330*; *Hopea dryobalanoides*: MH791329; *Shorea pachyphylla*: NC040966; *Shorea zeylanica*: NC040965; *Neobalanocarpus heimii*: NC041191; *Vatica mangachapoi*: NC041485; *Carica papaya*: NC010323); *Dillenia indica:* NC042740; *Schima argentea*: KY406780; *Franklinia alatamaha*: NC035692; *Stewartia calcicola*: NC035696; *Hartia laotica*: NC041509; *Camellia cuspidata:* NC022459; *Polyspora axillaris*: NC035645; *Laplacea fruticosa:* NC035700; *Euryodendron excelsum*: NC039178; *Clematoclethra scandens*: KX345299; *Actinidia arguta:* MG744576; *Viola mirabilis*: NC041582.
